# Effect of a diabetes-specific formula in non-diabetic inpatients with stroke: a randomized controlled trial

**DOI:** 10.1038/s41387-024-00292-4

**Published:** 2024-05-30

**Authors:** Juan J. López-Gómez, Esther Delgado García, David Primo-Martín, Mónica Simón de la Fuente, Emilia Gómez-Hoyos, Rebeca Jiménez-Sahagún, Beatriz Torres-Torres, Ana Ortolá-Buigues, Beatriz Gómez-Vicente, Juan F. Arenillas-Lara, Daniel A. De Luis Román

**Affiliations:** 1 Servicio de Endocrinología y Nutrición, Hospital Clínico Universitario de Valladolid, Valladolid, Spain; 2 Centro de Investigación en Endocrinología y Nutrición (IENVA), Universidad de Valladolid, Valladolid, Spain; 3 Department of Medicine, Universidad de Valladolid, Universidad de Valladolid, Valladolid, Spain; 4 Stroke Program, Department of Neurology, HCUV, Valladolid, Spain

**Keywords:** Pre-diabetes, Nutrition

## Abstract

**Background/objectives:**

In patients with acute stroke, the presence of hyperglycaemia has been associated with higher morbidity and less neurological recovery. The aim of the study was to evaluate the impact of a diabetes specific enteral nutrition (EN) formula on glycaemia, comorbidities and mortality in patients admitted with a first episode of stroke who received complete EN.

**Methods:**

This was a prospective randomised controlled trial. Patients with acute stroke did not have diagnosis of diabetes mellitus and required nasogastric tube feeding. This study has been registered with code NCT03422900. The patients were randomised into two arms: an isocaloric isoprotein formula (control group (CG), 27 patients) vs a diabetes-specific formula (low glycaemic index carbohydrates, fibre (80% soluble) and higher lipid content) (experimental group (EG), 25 patients). Pre-EN blood glucose, hyperglycaemia during EN treatment, HbA1c, insulin use, oral route recovery, length of stay (LOS) and mortality at 30 days were collected. The complications of enteral nutrition during admission were collected as well.

**Results:**

52 patients were included, 50% females, with an age of 77.44(11.48) years; 34 (65.4%) had ischaemic stroke, with a Rankin score of 0(0–2), and a National Institute of Health Stroke Scale (NIHSS) of 19 (15–22). In CG, there were more cases of hyperglycaemia on the 5th day post-NE (13(65%) vs7(35%), *p* < 0.01). CG showed an OR of 7.58(1.49–39.16) (*p* = 0.02) for the development of hyperglycaemia. There were no differences in LOS between groups (12(8.5) days vs 14(23) days, *p* = 0.19) or in the death rate (10(37%) vs 10(40%), *p* = 0.8), although differences were found in terms of oral route recovery (EG: 11(44%) patients vs CG: 5(18.5%) patients, *p* = 0.04) (OR (EG): 5.53(1.25–24.47); *p* = 0.02).

**Conclusions:**

The use of a diabetes-specific enteral formula in non-diabetic patients admitted with acute stroke reduced the risk of developing hyperglycaemia and improved the rate of oral route recovery.

Registered under ClinicalTrials.gov Identifier no. NCT03422900.

## Background and aims

Stroke is a neurological disorder derived from alterations in vascularisation in the brain. This pathology affects 15 million people year, causes 5.5 million deaths per year and results in the loss of 116 million years of quality of life [1]. In Spain, incidence of stroke is ~128 cases per 100.000 person years, it is the second cause of global death in the general population, with a mortality rate of 11% [2]. Stroke is a leading cause of death and disability worldwide [3].

There is a clear association between diabetes mellitus and vascular diseases [4]. It is well demonstrated that diabetes mellitus increases the risk of stroke. In addition, patients with diabetes have a double risk of suffering from stroke recurrence and an increase in complications and rehabilitation or functional recovery [5].

The development of hyperglycaemia after an episode of stroke can be related to a worse evolution of stroke-related damage [6]. In patients with diabetes mellitus, the hyperglycaemia produces an alteration in the recanalisation associated with a reduced reperfusion, a worse evolution of ischaemic penumbra area and direct damage in the tissues [7]. This alteration has shown an increase in mortality and a worse functional recovery in patients with ischaemic [8] or haemorrhagic stroke [9].

Oropharyngeal dysphagia is a symptom identified in up to 78% of patients in the acute post-stroke phase. This is one of the main causes of post-stroke mortality due to its association with complications such as malnutrition or aspiration pneumonia, which occurs in >20% of patients [10]. In these patients, medical nutrition therapy is usually required via an enteral route [11].

In patients with complete enteral nutrition, the incidence of hyperglycaemia can reach 30% [12]. In a previous study in 158 non-diabetic patients admitted by stroke in our hospital, 33% (52 patients) of non-diabetic patients with stroke and complete enteral nutrition developed stress hyperglycaemia and 19.1% (30 patients) developed hyperglycaemia related to the use of enteral nutrition [13].

Diabetes-specific formulas were developed as a method to avoid enteral nutrition-related hyperglycaemia in patients with diabetes. These formulas usually have a reduction of energy from carbohydrates and a replacement with energy from lipids or proteins; the use of carbohydrates with a low glycaemic index such as lactose or isomaltulose; and/or an increase in the amount of soluble fibre to decrease glucose absorption [14]. In addition, some of these formulas are enriched in monounsaturated (MUFA) and polyunsaturated fatty acids (PUFA) to have a benefit in the lipid profile [15].

Diabetes-specific formulas have shown an improvement in glycaemic control and lipid control in patients with diabetes. In 2005, Elia et al. concluded that the use of diabetes-specific formulas in the short- and long-term was associated with better glycaemic control related to standard formulas [16]. In 2019, Ojo et al. also showed better glycaemic control and an increase in HDL-cholesterol but no effect in other lipid parameters [17]. In 2020, Sanz Paris et al. studied diabetes-specific formulas that are high in MUFAs and concluded that these formulas can improve glucose control and metabolic risk factors among patients with diabetes [15]. Most of these studies are being developed in patients with diabetes and stress hyperglycaemia, but there are few studies that have compared diabetes-specific formulas with standard formulas in patients without diabetes.

In patients without diabetes who suffer a stroke, hyperglycaemia can be caused by several reasons: stress induced-hyperglycaemia, enteral nutrition, and acute treatments for stroke. As a result, medical nutrition therapy must be oriented to avoid this condition of hyperglycaemia. The use of diabetes-specific formulas in patients with stroke has shown an improvement in acute-term glycaemic control in severe acute ischaemic patients with non-insulin dependent diabetes and without diabetes [18]. However, there is no evidence of the use of these formulas in patients with stroke and no diabetes.

The purpose of this study was to evaluate the effect of a diabetes-specific nutritional formula on the development of hyperglycaemia in non-diabetic inpatients with recent stroke who require complete enteral nutrition through a nasogastric tube, as well as the effect of the use of this formula on the development of comorbidities, mortality and tolerance.

## Methods

### Study design

A randomised controlled clinical trial was developed in patients without diabetes mellitus admitted due to stroke and with an indication for nutritional support by nasogastric tube. We randomly assigned patients to receive either a diabetes-specific enteral formula or a standard formula.

The primary outcome of the study was the development hyperglycaemia associated with enteral nutrition formula during admission. The secondary outcomes were to observe the influence of enteral nutrition formula on comorbidity of stroke (oral route recovery, death, readmission and length of stay) and in complications of enteral nutrition formula.

This study was developed between January 2018 and September 2021 in patients admitted to the Stroke Unit, Department of Neurology of Clinic University Hospital of Valladolid. All patients signed informed consent before recruitment and randomisation.

The study was carried out in accordance with the Declaration of Helsinki and all procedures were approved by the Medical Research Ethics Committee (CEIm) of East Valladolid under code CASVE-NM-17-315. This clinical trial was registered in www.clinicaltrials.gov with code NCT03422900.

### Study subjects

Patients with a first-ever ischaemic or haemorrhagic stroke, without history of diabetes mellitus, admitted to the Stroke Unit of our hospital were included in the study. The patients had complete dysphagia and required total enteral nutrition via a nasogastric tube (for at least 7 days). The inclusion criteria were the start of enteral nutrition during the first 72 h after stroke and acceptance by the patient or their legal representative to participate in the study. The main reason to select 72 h was that there is no evidence of the use of early enteral nutrition by nasogastric tube could ameliorate the outcomes and if the patient has no dysphagia could be dangerous [3]. We select 72 h to ensure the stability of patient and the protocol to confirm dysphagia in these patients in our hospital.

The exclusion criteria were enteral nutrition not feasible; severe gastrointestinal pathology; diagnosed diabetes mellitus; current glycated haemoglobin (HbA1c) >6.5%; allergy or intolerance to ingredients of the formula; pregnant women; patients who required an Intensive Care Unit (ICU) stay; previous neurodegenerative disease; and did not sign the informed consent.

Randomisation was done by a randomisation seed generated by http://www.randomization.com/ with a block assignation AABB, ABAB, ABBA, BAAB, BBAA and BABA.

The investigators were aware of the allocation sequence and treatment allocation. The trial personnel who worked in hospital plant only administrate the enteral nutrition prescribed by investigators. There was no blinding because it was a trial in real clinical practice with the formulas commonly used in the hospital. Nevertheless, the investigator who made the randomization is not involved in the clinical follow-up of the patient. This investigator informed the allocation of treatment to the physician of clinical nutrition services that prescribed the indicated treatment. This physician is not involved in data collection or analysis. At the end of the study the physician who had followed the patient in a multidisciplinary team delivered a report of variables to the investigator that includes it in the database. Another investigator took care of the data analysis. In this way, the investigator who collected and analyzed the variables was not the physician who followed the patient.

There was no predefined stopping rule. In the development of study, we have seen a better glycaemia evolution in patients with diabetes-specific formula. For this reason, we consider the primary outcome was solved and all patients could benefit from the use of diabetes-specific formula.

### Medical nutrition therapy

A standard enteral nutrition formula (isocaloric, isoproteic formula without fibre) was compared with a diabetes-specific enteral formula (isocaloric and isoproteic formula with carbohydrates with a lower glycaemic index and the presence of mixed soluble and insoluble fibre) (Table 1).

**Table 1:** Distribution of calories, macronutrients and micronutrients of control and experimental formula.

Content/100 ml	Standard formula (control group)	Diabetes-specific formula (experimental group)
Caloric Content (kcal)	100	100
Macronutrients		
Proteins (g)	3.5	4.3
Proteins (%TCV)	15	17
Lipids (g)	3.4	3.9
* Saturated (g)*	*1.2*	1
* Monounsaturated (g)*	*1.5*	2
* Polyunsaturated (g)*	*0.7*	0.9
*MCT (g)*	*0.5*	-
Lipids (%TCV)	31	35
Carbohydrates (g)	13.5	11.3
Carbohydrates (%TCV)	54	45
Minerals		
Sodium (mg)	80	85
Chloride (mg)	75	65
Potassium (mg)	135	140
Calcium (mg)	70	90
Phosphate (mg)	60	70
Magnesium (mg)	17	20
Iron (mg)	1.1	1.5
Zinc (mg)	1.0	1.4
Copper (mg)	0.17	225
Iodine (mg)	13	34
Selenium (mg)	7	9,5
Manganese (mg)	0.24	0.45
Chrome (mg)	11	7.5
Molybdenum (mg)	13	11
Fluoride (mg)	0.13	0.18
Vitamins		
Vitamin A (μg)	110	85.4
Vitamin D (μg)	1.5	2.4
Vitamin K (μg)	7	20
Vitamin C (mg)	11	22
Thiamin (mg)	0.14	0.3
Riboflavin (mg)	0.17	0.33
Vitamin B6 (mg)	0.17	0.43
Niacin (mg)	1.7	1.5
Folic Acid (μg)	29	47
Vitamin B12 (mg)	0.38	0.6
Pantothenic acid (mg)	0.6	1.5
Biotin (mg)	4.6	3
Vitamin E (mg)	1.6	3
Choline (mg)	38	37
Other characteristics		
Osmolarity (mOsm/l)	236	160
Fibre (g)	0	1.5
* Soluble/Insoluble (%)*	-	80/20
*% TCV*	-	3

TCV: Total Caloric Value.

Energy requirements for patients were estimated by Harris Benedict equation with a stress factor of 1.2. The amount of enteral nutrition was calculated in the function of energy density of the formula and energy requirements of the patient.

Enteral nutrition was delivered with a continuous infusion pump for 24 h without rest.

### Study variables

Sociodemographic variables: Age (years), gender (male/female).

Clinical variables: Stroke type (ischaemic/haemorrhagic); stroke clinical severity (National Institutes of Health Stroke Scale (NIHSS)); Rankin scale.

Anthropometric Variables: Usual weight (kg); actual weight (kg); height (m); body mass index (kg/m^2^).

Metabolic variables:

- Biochemical: performed with a Cobas c-711 autoanalyser (Roche Diagnostics): glucose (mg/dL); creatinine (mg/dl); albumin (g/dL); C-reactive protein (CRP) (mg/dL), prealbumin (mg/dL); CRP/prealbumin ratio; glycaemia (mg/dL); sodium (mEq/l); potassium (mEq/l); urea (mg/dL); total cholesterol (mg/dL); and triglycerides (mg/dL).
- Diagnosis of hyperglycaemia: fasting plasma glucose >126 mg/dl before the beginning of enteral nutrition or glycaemia >180 mg/dl during the administration of the enteral formula.
- Capillary blood glucose (Abbott^®^ Lifestyle Measurer): this was considered the average of capillary glycaemia (mg/dl) every 8 h for 7 days, from the start of enteral nutrition until discharge from hospital.

Gastrointestinal Tolerance: diarrhoea, constipation, gastric emptying, abdominal distension, presence of vomiting. All of these variables were collected daily in the form of a dichotomous variable yes/no by the same investigator.

Complications: length of stay (days); mortality during admission; readmission; presence of pneumonia; dysphagia recovery (negative method of clinical volume-viscosity test (MECV_V)) [19].

### Statistical analysis

The data were stored in a database on the statistical package SPSS 23.0 (SPSS Inc. Il, USA) with an official license from the University of Valladolid. An analysis of normality of the continuous variables was performed with the Kolmogorov-Smirnov test.LIe.

The calculated sample size was 150 patients (75 per branch with 10% loss). The study had to be stopped at 60 patients because the primary aim was achieved, and we could not continue due to ethical considerations, these considerations were to obtain the main outcome in patients with diabetes-specific formula, so all the patients could benefit from the use of diabetes-specific formula.

Continuous variables were expressed as mean (standard deviation), parametric variables were analysed with the unpaired and paired *t*-Student test, and non-parametric variables with the Friedman, Wilcoxon, K Kruskal, and *U*-Mann Whitney tests. If it was necessary to compare variables in more than two groups, the ANOVA *U* test (with the Bonferroni post-hoc test) was used. The analysis of variables at the different times of the study was performed using multivariable analysis of variance (MANOVA).

A multivariable analysis was performed adjusted by variables which show differences between groups in a descriptive analysis and with variables which can influence glycaemia.

Qualitative variables were expressed as percentages (%) and analysed using the Chi-square test (with Fisher and Yates corrections when necessary). A *p*-value < 0.05 was considered significant.

## Results

**Fig. 1:**
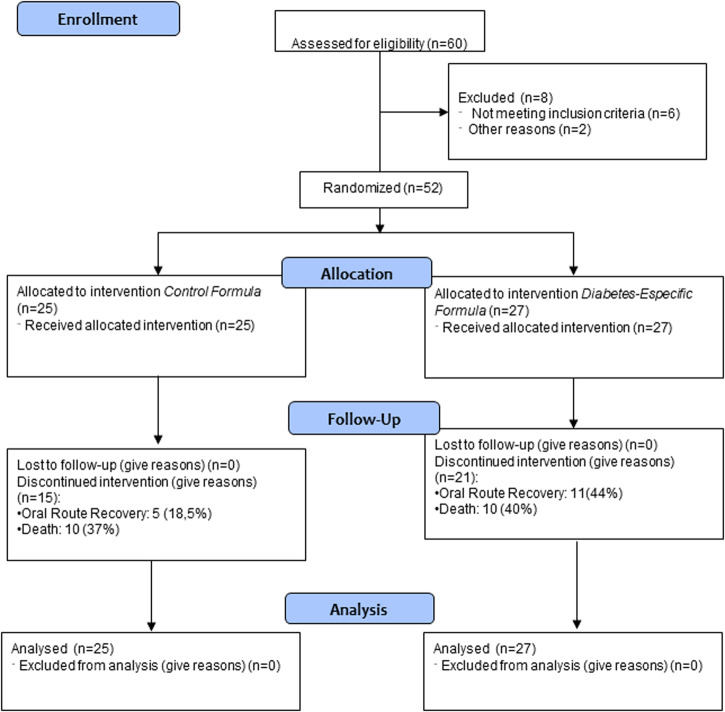
Flow chart.

Sixty patients were assessed for eligibility, 8 patients were excluded, and 52 patients were randomised (25 patients to the intervention group and 27 patients to the control group) (Fig. 1).

### Sample Description

There were no differences between variables in both groups except for natremia and stroke type (ischaemic/haemorrhagic) (Table 2).

**Table 2:** Differences in variables at the start of the study in experimental and control group.

	TOTAL	CONTROL	EXPERIMENTAL
Gender (M/F)	26 (50%)/ 26 (50%)	11 (44.5%)/ 14 (55.6%)	15 (56%)/ 12 (44%)
BMI (kg/m^2^)	26.6 (4.0)	26.9 (4.6)	26.3 (3.3)
Calf Circunference (cm)	33.0 (4.2)	32.9 (4.4)	33.1 (4.2)
SGA (A/B/C)	28 (53.8%)/13 (25%)/10 (19.2%)	12 (48.1%)/ 7 (25.9%)/ 6 (22.2%)	16 (60%)/6 (24%)/5 (16%)
Age (years)	77.4 (11.5)	78.9 (9.7)	75.9 (13.2)
RANKIN scale	0 (0–2)	0 (0–0)	0 (0–2.5)
NIHSS	17.6 (6.4)	16.9 (7.1)	18.3 (5.6)
Glomerullar Filtration (ml/min)	84.7 (29.7)	84.2 (35.9)	85.2 (22.2)
Na (mg/dL)*	142.9 (3.9)	144.1 (3.4)	141.7 (4.2)
K (mg/dL)	4.0 (0.4)	3.9 (0.5)	4.1 (0.4)
Plasma Insulin (mg/dL)	15.5 (11.7)	14.2 (7.1)	16.6 (14.7)
HbA1c (%)	5.6 (0.4)	5.6 (0.4)	5.6 (0.4)
Glycaemia (mg/dL)	113.7 (21.8)	115.7 (22.5)	111.5 (21.3)
Total Cholesterol (mg/dL)	167.8 (38.8)	174.1 (41.0)	160.8 (35.7)
Triglycerides (mg/dL)	98.6 (33.8)	101.7 (38.4)	95.2 (28.4)
Length of stay (days)	15.0 (11.1)	12 (7–30)	14 (7–30)
Stroke type (Ischaemic/hemorragic)*	34 (65.4%)/18 (34.6%)	13 (51.9%)/12 (48.1%)	22 (80%)/ 5 (20%)

*BMI* Body Mass Index, *M* Male, *F* Female, *SGA* Subjective Global Assessment, *Na* sodium, *K* potassium, *NIHSS* National Institutes of Health Stroke Scale. *p-value < 0.05.

### Evaluation of glycaemic parameters during the admission

Fasting venous glucose levels after 5 days of enteral nutrition was 133 (118–160.5) mg/dl. The values were significantly higher in the control group (Control (C): 150.5 (132.25–173.5) mg/dl vs. Experimental (E): 121 (113.5–142) mg/dl; *p*-value = 0.02).

Average capillary blood glucose levels after 7 days of enteral nutrition were 130.58 (22.6) mg/dl. No statistically significant differences were found between the two groups (C: 132.96 (22.56) mg/dl vs. E: 127.79 (22.83) mg/dl; *p*-value = 0.43).

In addition, the absolute number of participants who developed hyperglycaemia (more than two episodes) after 5 days of enteral nutrition (cut-off point the value of 140 mg/dl and 180 mg/dl) had a higher rate of hyperglycaemia in the control group using the cut-off point 140 (CONTROL: 16 patients (65%); EXPERIMENTAL: 9 patients (35%); *p* < 0.05) and at the cut-off point 180 mg/dl (CONTROL: 16 patients (64.7%); EXPERIMENTAL: 9 patients (35.3%); *p* < 0.05).

A multivariable analysis was performed to assess whether the use of the control formula is an independent risk factor for the development of hyperglycaemia ( > 140 mg/dl) after 5 days of enteral nutrition. The use of a control formula was an independent risk factor for the development of hyperglycaemia (OR: 7.58 (1.47–39.16); *p*-value: 0.02), adjusted by type of stroke, age, use of corticosteroids, NIHSS value and sodium levels (Table 3).

**Table 3:** Multivariate Analysis for the development of hyperglycaemia 5 days after start enteral nutrition related to control formula, stroke type, age and plasma sodium; NIHSS: National Institutes of Health Stroke Scale.

Hyperglycaemia (5 days)	OR	IC 95%	*p*-value
Control Formula	7.78	(1.43–42.19)	0.02
Stroke type	5.17	(0.66–40.31)	0.12
Age	1.07	(0.98–1.16)	0.14
NIHSS	1.09	(0.94–1.26)	0.23
Use of corticosteroids	1.34	(0.08–21.39)	0.84
Plasma Sodium	1.00	(0.79–1.26)	0.98

The use of subcutaneous basal insulin to reach normal glycaemia during admission was needed in 13 patients (25%) (9 (33%) from the control group vs. 4 (16%) from the experimental group, with no statistical difference between groups).

**Fig. 2:**
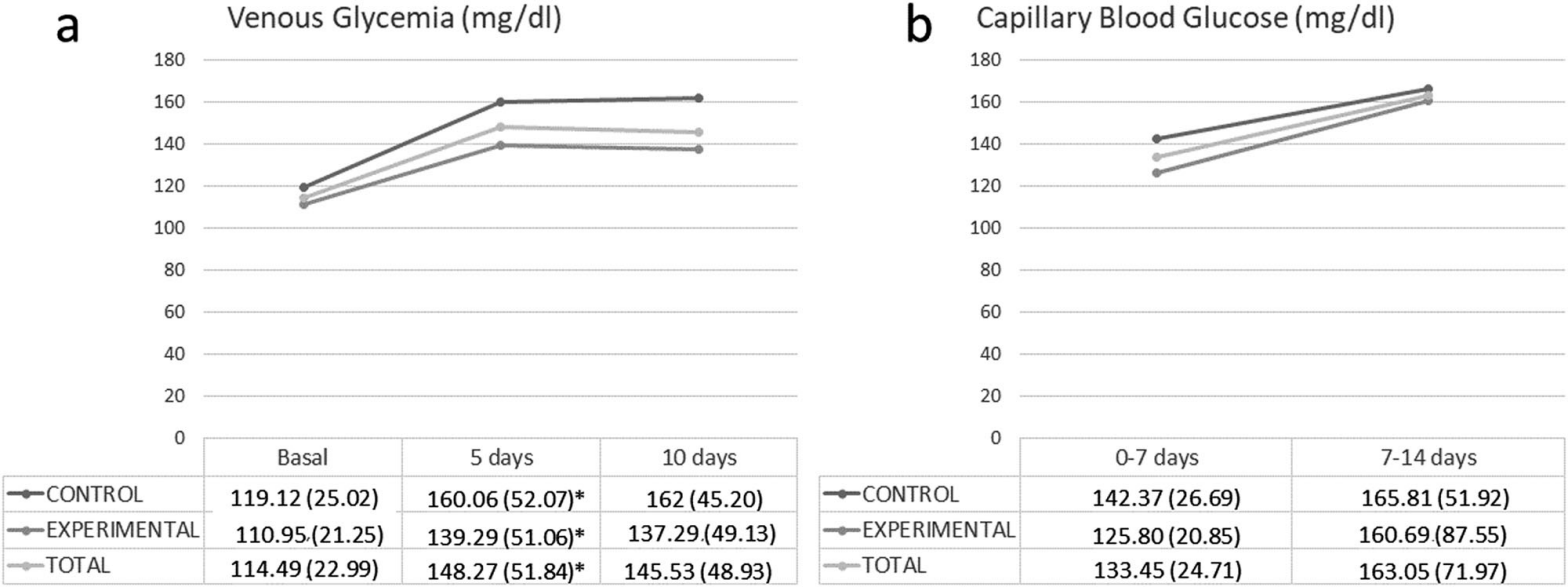
Differences in glycaemia changes between groups (control, experimental and total) with two different methods. **a** Basal venous glycaemia, 5 days glycaemia and 10 days glycaemia, and in **b** average capillary blood glycaemia at 0–7 days and 7–14 days. malization and correction for chance.

The evolution of the venous glycaemia of the subjects was compared between groups (Fig. 2). It was observed that the glycaemia in the experimental group was lower at the three determined cut-off points (baseline, at 5 days, and at 10 days). On the other hand, capillary glycaemia tended to normalise 14 days after starting enteral nutrition. There were differences in the control group and total sample but there were no differences in the experimental group (Fig. 2).

It was observed a significant decrease in total cholesterol in both groups 5 days after the start of enteral nutrition (CONTROL: Start: 171.84 (41.44) mg/dL; 5 days: 132.95 (24.21) mg/dL; *p* < 0.01; EXPERIMENTAL: Start: 170.61 (32.77) mg/dL; 5 days: 135.39 (20.55); *p* < 0,01); at 10 days after the start of enteral nutrition there was a significant decrease in control group (CONTROL: Start: 171.84 (41.44) mg/dL; 10 days: 135.67 (29.10) mg/dL; *p* < 0.01; EXPERIMENTAL: Start: 170.61 (32.77) mg/dL; 10 days: 147.95; *p* = 0.07). The decrease in total cholesterol was more striking in control group (CONTROL: 34.48 (11.63) %; EXPERIMENTAL: 11.33 (23.35) %; *p* = 0.04). There were no significant differences in level of triglycerides in both groups.

### Evaluation of comorbidities

Patients receiving the experimental formula showed greater recuperation of the oral route than the control group. There were no differences in terms of length of hospital stay, readmission or death (Table 4).

**Table 4:** Differences in outcomes during admission (recovery oral route and length of stay) and 3 months after intervention (readmission and exitus) between control and experimental group.

	Total	Control	Experimental	*p*-value
Recovery oral route	16 (30.8%)	5 (18.5%)	11 (44%)	<0.05
Length of stay > 14 days	17 (32.7%)	6 (22.2%)	11 (44%)	0.09
Readmission	11 (21.2%)	6 (22.2%)	5 (20.8%)	0.90
Exitus	20 (38.5%)	10 (37%)	10 (40%)	0.83

A multivariable analysis was performed to assess risk factors for the recovery of the oral route at discharge and as independent variables: the use of the experimental formula (diabetes-specific), age and type of stroke. It was observed that the use of a diabetes-specific formula increased the probability of recovery of the oral route at hospital discharge (OR: 6.26 (95% CI: 1.22–32.17); *p*-value = 0.03) adjusted by NIHSS (OR: 0.89 (95%: 0.79–0.99); *p*-value < 0.05); age (OR: 0.98 (95% CI: 0.93–1.04); *p*-value = 0.57) and type of stroke (OR: 2.3 (95%: 0.46–11.59); *p* = 0.31).

They were evaluated as independent risk factors for mortality: type of formula, age, NIHSS and type of stroke. There was no statistical relationship. On the other hand, the length of stay > 14 days did not show any association with the variables age, type of stroke or type of formula.

### Evaluation of complications

The rate of digestive complications was evaluated. There was an increase in the development of diarrhoea in the experimental group. There were no significant differences for the rest of the digestive complications (Table 5). No statistically significant differences were observed for other admission complications (Table 5).

**Table 5:** Differences in complications during admission (30 days of intervention) between control and experimental group.

	TOTAL	CONTROL	EXPERIMENTAL	*p*-value
Digestive Complications				
Abdominal Distension	1 (1.9%)	0	1 (4%)	0.30
Vomiting	3 (5.8%)	1 (3.7%)	2 (8%)	0.51
Constipation	4 (7.7%)	3 (11.1%)	1 (4%)	0.34
Diarrhoea	5 (9.6%)	0	5 (20%)	0.02
Admission Complications				
Bronchial Aspiration	11 (21.2%)	5 (18.5%)	6 (24%)	0.63
Readmission	11 (21.2%)	5 (20.8%)	6 (22.2%)	0.90
Death	20 (38.5%)	10 (37%)	10 (40%)	0.83

## Discussion

In non-diabetic patients who are admitted for stroke and require complete enteral nutrition via an enteral route, the use of a diabetes-specific formula decreased the rate of hyperglycaemia associated with enteral nutrition. These patients also showed better glycaemic control during the admission and better recovery of the oral route.

The age of patients ranged from 75–80 years, like the median age of patients with stroke in most studies. The incidence of stroke increased with age with a high rate in older patients [20]. Most of the patients presented a normal nutritional status at admittance assessed by subjective global assessment; the mean BMI showed an overweight state. These data are like those of other studies which assessed malnutrition in patients with stroke at admission. Sato et al. observed that 57% of patients admitted to a Stroke unit have a normal nutritional status assessed by GLIM criteria [21]. If we observe functional status prior to stroke in patients, the modified RANKIN scale shows a very slight disability in our sample related to the age of patients; the rate of great disability in our patients were very low.

There was also a difference between the type of stroke, with an increase in ischaemic stroke in the experimental group, these differences could be based on chance and must not influence results because there are no data on differences in the development of hyperglycaemia related to the type of stroke [22]. Hyperglycaemia and diabetes are related to worse events in patients with ischaemic or haemorrhagic stroke. However, there are no clear effects of hyperglycaemia on differences in events related to the type of stroke [22].

The use of diabetes-specific formulas have been studied in patients with diagnosed diabetes mellitus with better control in fast glucose and glycated haemoglobin, as Ojo et al. showed in a recent metanalysis [17]. However, the effect on the development of hyperglycaemia in patients with no previous diagnosis of diabetes is still unclear. Sanz et al. showed that high monounsaturated fatty acid diabetes-specific formulas can ameliorate the glycaemic control in patients with diabetes or stress-induced hyperglycaemia [15]. Our study has shown that the use of this type of formulas in patients admitted for stroke can decrease the development of hyperglycaemia induced by enteral nutrition. Another similar study by Shao et al. in patients with ischaemic stroke with complete enteral nutrition by nasogastric tube proved that the use of a diabetes-specific formula may improve acute-term glycaemic control in severe acute ischaemic stroke patients, but there were no differences in glycaemic variability control. The main differences with our study were the selection of patients with or without diabetes, except insulin-dependent diabetes [18]. Our study population only included patients without any type of previous diagnosed or undiagnosed diabetes and the patients can be either ischaemic or haemorrhagic stroke.

Dysphagia is a frequent complication after a stroke. A recent study has estimated that it occurs in >20% of stroke patients and persists in >50%. The severity of the stroke, more than the location, is what determines the appearance of dysphagia. Dysphagia deteriorates nutritional status and increases the risk of aspiration pneumonia in >20% of patients, which causes death in 20% of patients in the year following the stroke [23]. In our study, patients in the interventional group had an increased probability (OR: 5.53) of recovery of the oral route. As seen in recent studies, the development of hyperglycaemia is related to non-recovery of the oral route in these patients. An intervention which avoid the increase of glycaemia can improve the dysphagia [13]. Our trial shows that the diabetes-specific formula produces a lower rate of hyperglycaemia and, therefore, greater recovery from the oral route. However, it is necessary to design studies directed to study the oral route recovery to better assess the effect of the control of glycaemia and the use of a diabetes-specific formula.

There were no significant differences in the percentage of deaths in both groups. Hospital mortality due to stroke has decreased from 1970–2008 from 35.9%–19.8%. Some of the factors associated with an increased risk of mortality after a stroke are the following: age, the initial severity of the stroke, gender, race, previous functional status, hyperthermia, hyperglycaemia, high or low blood pressure and previous cardiovascular diseases [24]. In our trial, mortality was higher, around 40%, due to the extension of stroke that involved the ability to swallow, and the advanced age (77 years) of the patients included in the study. The need for a nasogastric tube is usually linked to a greater clinical severity and/or to the development of severe clinical complications [25]. There were no differences between groups, although there was better glycaemic control in the experimental group.

An increase in the development of diarrhoea in the experimental group was shown, probably related to the enteral formula. Diarrhoea is the most frequent gastrointestinal complication in patients receiving enteral nutrition. In general, the prevalence is around 30% in hospitalised patients, reaching 80% in those who are in the intensive care unit (ICU) [26]. The causes of diarrhoea associated with enteral nutrition are varied. The main causes are high osmolarity of the formula and the fibre content. In the experimental group, osmolarity of the formula was lower than in the control group but there was a mixed fibre content with 80% soluble fibre and 20% insoluble fibre. These characteristics may have influenced the development of diarrhoea in the experimental group. In the study by León et al., which compared two specific diabetes enteral formulas (low carbohydrate and high lipids content vs. high carbohydrate diet), the high carbohydrate group showed a significantly higher incidence of diarrhoea, as in our study [27]. However, another study performed by De Luis et al. showed no differences in diarrhoea between high or low doses of a diabetes-specific formula. This situation can be related to the use of the formula as oral nutritional supplementation and not in a complete route [28].

The main strength of the study is the type of study as a randomised controlled trial with two different enteral formula. Otherwise, the population selected, as the use of a diabetes-specific formula has mainly been studied in patients with diagnosed diabetes and its prescription in patients with a risk of development of hyperglycaemia has a lack of evidence. On the other hand, in addition to the reported effect on glycaemia, differences were observed in outcome variables such as recovery of the oral route in those fed with the specific formula for diabetes, a condition that could be studied in subsequent trials and well-designed trials.

The main limitations of the study were the different type of stroke studied that could interfere with the results. However, as we have adjusted multivariable analysis with this condition, the type of stroke seems not to influence the main objective of the study on glycaemic control and secondary outcomes as oral route recovery. Another limitation was that the calculated sample size was 150 patients, but the study had to be stopped at 60 because it had accomplished the primary aim, and we could not continue due to ethical considerations. We think that more striking results on *p*-value must be a better guarantee for reducing possible false positive results. Nevertheless, the use of diabetes-specific formulas has a long experience in control hyperglycaemia in diabetic patients in critic and non-critic condition. There is less experience in non-diabetic patients but as we can see in these patients, the behaviour was similar in glycaemia, and we see secondary aims as oral route recovery in patients with an adequate control of glucose metabolism. These two reasons lead us to plant the use of a more specific formula in all patients. On the other hand, there was no blinding due to the use of usual formulas of the hospital in real clinical practice; we think that these characteristics did not influence the trial result because the use of diabetic enteral formulas is clear in patients with diabetes, but this not so clear in patients with diagnosed diabetes. On the other hand, the separation between investigators who allocated patients to the different groups of study and the physicians that followed the patients in their clinical route avoid the possible bias of not blinding the study.

The main clinical consequences of the development of this study are the planification of use diabetes-specific formulas (enteral formulas with low glycaemic index carbohydrates, monounsaturated fatty acids and mixed fibre) in non-diabetic patients with ischaemic or hemorragic stroke during admission with a low rate of complications.

These formulas have shown a decrease in hyperglycaemia, but it is necessary to develop studies to show whether the use of these formulas can improve the evolution of stroke. These studies can improve the results using continuous glucose monitoring systems to evaluate the glycaemic variability and its relationship with the secondary outcomes of the evolution of stroke. Another point to investigate is the different evolution of stroke by imaging techniques in relation with glycaemia evolution.

## Conclusions

The use of a specific-diabetic formula in non-diabetic patients with stroke and complete enteral nutrition by a nasogastric tube showed a lower development of hyperglycaemia episodes compared to an isocaloric isoprotein formula. The use of a diabetes-specific formula produced lower venous and capillary glycaemia compared to the control formula.

In patients treated with a diabetes-specific formula, there was an increased recovery of the oral route, which could be related to the less frequent development of hyperglycaemia. No differences between groups were observed in the mean hospital stay, readmissions or percentage of deaths.

It is necessary to develop new studies on the use of diabetes-specific formulas in non-diabetic patients to assess clinical outcomes of the nutritional intervention. The effect of glycaemic control in patients without diabetes treated with a diabetes-specific formula may help to create some lines of investigation into the effect of glycaemic control using the new continuous glucose monitoring devices. Also, given the results obtained for oral route recovery, it would be interesting to develop studies with this aim as a principal objective.

## Data Availability

The datasets generated during and/or analysed during the current study are not publicly available due to confidentiality of clinical records but are available from the corresponding author on reasonable request.
